# Bis[5-(pyridin-2-yl)pyrazine-2-carbo­nitrile-κ^2^ *N* ^4^,*N* ^5^](trifluoro­acetato-κ*O*)silver(I)

**DOI:** 10.1107/S1600536812040846

**Published:** 2012-10-06

**Authors:** Jing Li, Xiao Cuan Jia, Jun Qi, Yuan Yuan Liu, Yu Ding

**Affiliations:** a Tianjin Entry–Exit Inspection and Quarantine Bureau, Tianjin 300201, People’s Republic of China; b Disease Hospital of TianJin City, Tianjin 300201, People’s Republic of China

## Abstract

In the asymmetric unit of the title compound, [Ag(C_10_H_6_N_4_)_2_(CF_3_CO_2_)], there two mononuclear but slightly different complex units. In each, two κ^2^ *N*:*N*-chelating 5-(pyridin-2-yl)pyrazine-2-carbonitrile ligands surround the Ag^I^ atom, giving an N_4_O square-pyramidal coordination geometry with one trifluoro­acetate O atom at the apex. The difference between the two lies in the Ag—N bond lengths: in one complex, three normal [range 2.272 (5)–2.552 (5) Å] and one long [2.706 (4) Å] and in the second, two normal [2.254 (5) and 2.290 (5) Å] and two long [2.647 (5) and 2.675 (5) Å] are present. Short inter­molecular F⋯F contacts [2.586 (4) Å] and weak π–π stacking inter­actions [minimum ring centroid separation 3.836 (5) Å] between pyridyl and pyrazinyl rings connect the complex units, forming columns which extend along the *b*-axis direction.

## Related literature

For metal complexes with pyridyl-based ligands, see: Wang *et al.* (2009 ▶); O’Keeffe & Yaghi (2012 ▶); Choudhury *et al.* (2002 ▶). For complexes with 5-(pyridin-2-yl)pyrazine-2-carbonitrile, see: Wang *et al.* (2010 ▶); Zhang & Yang (2011 ▶). For van der Waals radii, see: Pauling (1960 ▶).
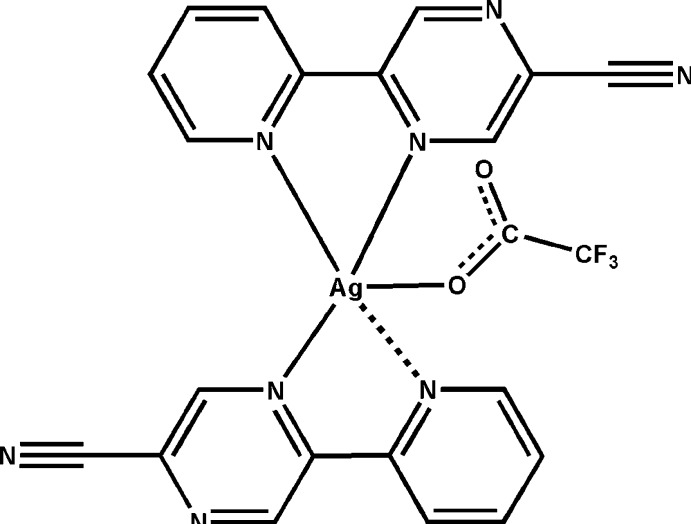


## Experimental

### 

#### Crystal data

[Ag(C_10_H_6_N_4_)_2_(C_2_F_3_O_2_)]
*M* *_r_* = 585.27
Orthorhombic, 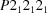

*a* = 12.5237 (16) Å
*b* = 14.9638 (18) Å
*c* = 23.845 (3) Å
*V* = 4468.6 (10) Å^3^
*Z* = 8
Mo *K*α radiation
μ = 0.97 mm^−1^
*T* = 298 K
0.41 × 0.31 × 0.29 mm

#### Data collection

Bruker APEXII CCD area detector diffractometer
Absorption correction: multi-scan (*SADABS*; Bruker, 2007 ▶) *T*_min_ = 0.861, *T*_max_ = 1.000
24472 measured reflections
7881 independent reflections
6180 reflections with *I* > 2σ(*I*)
*R*_int_ = 0.035

#### Refinement

*R*[*F* ^2^ > 2σ(*F* ^2^)] = 0.044
*wR*(*F* ^2^) = 0.129
*S* = 1.02
7881 reflections
649 parameters
10 restraints
H-atom parameters constrained
Δρ_max_ = 0.97 e Å^−3^
Δρ_min_ = −0.64 e Å^−3^
Absolute structure: Flack (1983 ▶), 3488 Friedel pairs
Flack parameter: −0.10 (4)

### 

Data collection: *APEX2* (Bruker 2007 ▶); cell refinement: *APEX2* and *SAINT* (Bruker 2007 ▶); data reduction: *SAINT*; program(s) used to solve structure: *SHELXS97* (Sheldrick, 2008 ▶); program(s) used to refine structure: *SHELXL97* (Sheldrick, 2008 ▶); molecular graphics: *SHELXTL* (Sheldrick, 2008 ▶); software used to prepare material for publication: *SHELXTL* and *PLATON* (Spek, 2009 ▶).

## Supplementary Material

Crystal structure: contains datablock(s) I, global. DOI: 10.1107/S1600536812040846/zs2235sup1.cif

Structure factors: contains datablock(s) I. DOI: 10.1107/S1600536812040846/zs2235Isup2.hkl

Additional supplementary materials:  crystallographic information; 3D view; checkCIF report

## Figures and Tables

**Fig. 1.**
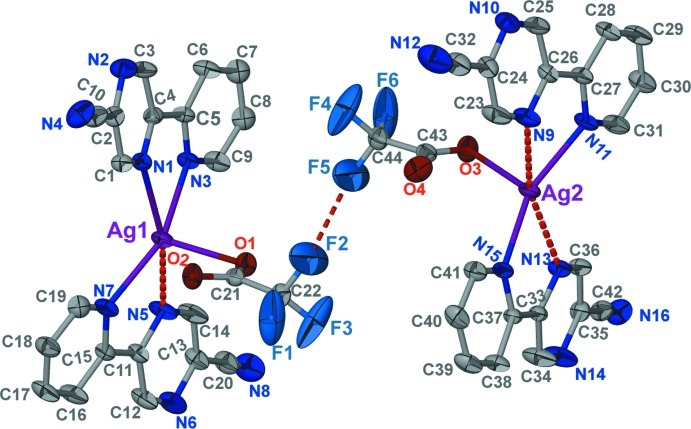
The atom-numbering scheme for the two independent complex units in the asymmetric unit of the title complex. Displacement ellipsoids are drawn at the 30% probability level and the intermolecular F···F interaction is shown as a dashed line. All H atoms are omitted for clarity.

**Fig. 2.**
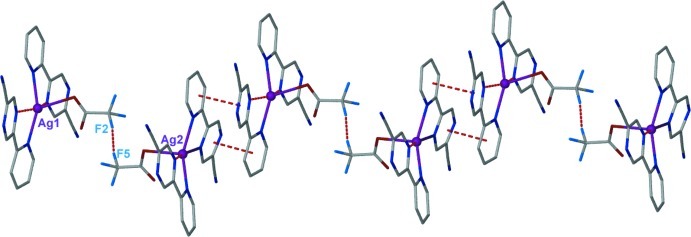
The inter-unit F···F and π···π interactions between the mononuclear units extending along b. The purple balls indicate Ag atoms. All H atoms are omitted.

**Fig. 3.**
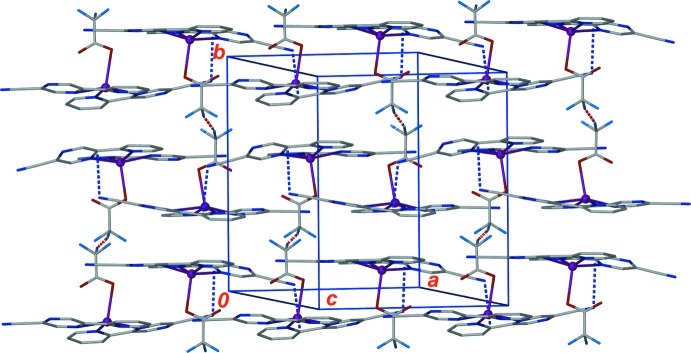
The packing structure of the title complex. Dashed-lines represent F···F and N(acetonitrile)···C(pyrazine) contacts.

## supplementary crystallographic information

### Comment

Multidentate ligands are widely used to construct diverse metal-organic frameworks with attractive topological structures and interesting properties (Wang *et al.*, 2009; O'Keeffe & Yaghi, 2012). 5-(2-pyridyl)pyrazine-2-carbonitrile is a new member of the family of pyridyl-based multidentate ligands, several mononuclear silver(I) complexes of which were reported recently (Wang *et al.*, 2010; Zhang & Yang, 2011). In the present context, we report the structure of a Ag^I^ complex with 5-(2-pyridyl)pyrazine-2-carbonitrile, the title compound [Ag(C_10_H_6_N_4_)_2_ (CF_3_CO_2_)] (Scheme 1).

As shown in Fig. 1, there are two independent but slightly different mononuclear units (associated with Ag1 and Ag2) in the asymmetric unit of the title complex. For Ag1, a pair of chelating κ^2^*N,N* 5-(2-pyridyl)pyrazine-2-carbonitrile ligands surround the Ag center to form a N_4_O-pyramidal coordination geometry, with a trifluoroacetate O bonding at the vertex site. The four N donors exibit different Ag—N bond lengths, with the Ag1—N5 [2.706 (4) Å] longer than the others [2.272 (5)–2.552 (5)Å]. The Ag2 unit exhibits a similar N_4_O-geometry to that for Ag1. However, both Ag2—N9 [2.647 (5) Å] and Ag2—N13 [2.675 (5) Å] are both longer than the other two Ag—N bonds [2.254 (5)–2.290 (5)Å]. This N_4_O-pyramidal coordination geometry is comparable to that found in the previously reported Ag complex with 5-(2-pyridyl)pyrazine-2-carbonitrile [Ag(C_10_H_6_N_4_)_2_]NO_3_ (Zhang & Yang, 2011). Interestingly, the two mononuclear units in the title complex are interconnected by a short F2···F5 interaction [2.586 (4) Å], giving a dimer. This F···F separation is much shorter than the sum of the van der Waals radii (2.70 Å) (Pauling, 1960), and the distance found in 1-(4-fluorophenyl)-2-phenyl-6-methoxy-1,2,3,4-tetrahydroisoquinoline [2.778 (2) %A] (Choudhury *et al.*, 2002).

In the present complex, F···F-connected dimers are interconnected through weak π···π stacking interactions between adjacent pyridyl rings and pyrazinyl rings, forming columns along *b* (Fig.2). The minimum distance between *Cg*1 (N1—C1—C2—N2—C3—C4) and *Cg*2^i^ (C37—C38—C39—C40—C41—N15) is 3.836 (5) Å, while that between *Cg*3 (C5—C6—C7—C8—C9—N3) and *Cg*4^i^ (N13—C33—C34 —N14—C35—C36) is 3.975 (3) Å [symmetry code: (i) *x*, *y* + 1, *z*]. Present within the columns are found short nonbonding N···C contacts between the acetonitrile N atoms and pyrazinyl C atoms (Fig. 3). These are N4···C37^ii^ [3.246 (5) Å] and N16···C4^iii^ [3.148 (4) Å] [symmetry codes: (ii) -*x*, *y* + 1/2, -*z* + 1/2; (iii) -*x* + 1, *y* - 1/2, -*z* + 1/2].

### Experimental

The 5-(2-pyridyl)-2-cyanopyrazine was obtained from a commercial source. This ligand (36.2 mg, 0.2 mmol) and AgCF_3_CO_2_ (22 mg, 0.1 mmol) were mixed and dissolved in 5 ml of acetonitrile with stirring at room temperature, giving a clear solution. After 2 hours, this solution was filtered, and the clear filtrate was allowed to stand for about 3 weeks, yielding yellow block-like crystals (32.8 mg, 56% yeild).

### Refinement

All the H atoms were discernible in the difference electron density maps. Nevertheless, the hydrogen atoms were placed in idealized positions and allowed to ride on the carrier atoms, with C—H = 0.93 Å and *U*_iso_(H) = 1.2*U*_eq_(C). The high *U*_eq_ value compared to neighbors for the C22 and C44 atoms of the trifluoroacetate ligand can be ascribed to the large thermal vibration of the tail of the anion at room temperature.

### Crystal data

**Table:**

[Ag(C_10_H_6_N_4_)_2_(C_2_F_3_O_2_)]	*F*(000) = 2320
*M**_r_* = 585.27	*D*_x_ = 1.740 Mg m^−^^3^
Orthorhombic, *P*2_1_2_1_2_1_	Mo *K*α radiation, λ = 0.71073 Å
Hall symbol: P 2ac 2ab	Cell parameters from 254 reflections
*a* = 12.5237 (16) Å	θ = 1.6–25.0°
*b* = 14.9638 (18) Å	µ = 0.97 mm^−^^1^
*c* = 23.845 (3) Å	*T* = 298 K
*V* = 4468.6 (10) Å^3^	Block, yellow
*Z* = 8	0.41 × 0.31 × 0.29 mm

### Data collection

**Table:**

Bruker APEXII CCD area detector diffractometer	7881 independent reflections
Radiation source: fine-focus sealed tube	6180 reflections with *I* > 2σ(*I*)
Graphite monochromator	*R*_int_ = 0.035
ω–scans	θ_max_ = 25.0°, θ_min_ = 1.6°
Absorption correction: multi-scan (*SADABS*; Bruker, 2007)	*h* = −14→9
*T*_min_ = 0.861, *T*_max_ = 1.000	*k* = −17→17
24472 measured reflections	*l* = −27→28

### Refinement

**Table:**

Refinement on *F*^2^	Secondary atom site location: difference Fourier map
Least-squares matrix: full	Hydrogen site location: inferred from neighbouring sites
*R*[*F*^2^ > 2σ(*F*^2^)] = 0.044	H-atom parameters constrained
*wR*(*F*^2^) = 0.129	*w* = 1/[σ^2^(*F*_o_^2^) + (0.0674*P*)^2^ + 4.5918*P*] *P* = (*F*_o_^2^ + 2*F*_c_^2^)/3
*S* = 1.02	(Δ/σ)_max_ = 0.005
7881 reflections	Δρ_max_ = 0.97 e Å^−^^3^
649 parameters	Δρ_min_ = −0.64 e Å^−^^3^
10 restraints	Absolute structure: Flack (1983), 3488 Friedel pairs
Primary atom site location: structure-invariant direct methods	Flack parameter: −0.10 (4)

### Special details

**Table:**

Geometry. All esds (except the esd in the dihedral angle between two l.s. planes) are estimated using the full covariance matrix. The cell esds are taken into account individually in the estimation of esds in distances, angles and torsion angles; correlations between esds in cell parameters are only used when they are defined by crystal symmetry. An approximate (isotropic) treatment of cell esds is used for estimating esds involving l.s. planes.
Refinement. Refinement of F^2^ against ALL reflections. The weighted R-factor wR and goodness of fit S are based on F^2^, conventional R-factors R are based on F, with F set to zero for negative F^2^. The threshold expression of F^2^ > 2sigma(F^2^) is used only for calculating R-factors(gt) etc. and is not relevant to the choice of reflections for refinement. R-factors based on F^2^ are statistically about twice as large as those based on F, and R- factors based on ALL data will be even larger.

### Fractional atomic coordinates and isotropic or equivalent isotropic displacement parameters (Å^2^)

**Table:**

	*x*	*y*	*z*	*U*_iso_*/*U*_eq_	
Ag1	0.26811 (4)	0.58972 (3)	0.15292 (2)	0.05751 (16)	
N1	0.1564 (4)	0.6017 (4)	0.2421 (2)	0.0516 (13)	
N2	0.0587 (5)	0.6226 (5)	0.3462 (3)	0.0758 (17)	
N3	0.3706 (4)	0.6293 (4)	0.2287 (2)	0.0527 (14)	
N4	−0.1913 (6)	0.5499 (5)	0.3209 (3)	0.093 (2)	
N5	0.3874 (5)	0.6246 (6)	0.0607 (2)	0.079 (2)	
N6	0.4762 (5)	0.6152 (6)	−0.0451 (3)	0.085 (2)	
N7	0.1688 (5)	0.6166 (5)	0.0750 (2)	0.0601 (16)	
N8	0.7394 (5)	0.6236 (7)	−0.0189 (3)	0.109 (3)	
O1	0.2973 (3)	0.4296 (3)	0.1556 (3)	0.0661 (12)	
O2	0.1194 (4)	0.4314 (3)	0.1498 (3)	0.0742 (14)	
C1	0.0527 (5)	0.5835 (5)	0.2491 (3)	0.0551 (16)	
H1A	0.0121	0.5654	0.2185	0.066*	
C2	0.0051 (5)	0.5914 (5)	0.3012 (3)	0.0559 (17)	
C3	0.1610 (7)	0.6397 (6)	0.3375 (3)	0.072 (2)	
H3A	0.2015	0.6602	0.3676	0.087*	
C4	0.2113 (6)	0.6289 (4)	0.2861 (3)	0.0503 (16)	
C5	0.3309 (6)	0.6352 (5)	0.2808 (3)	0.0512 (16)	
C6	0.3954 (7)	0.6446 (6)	0.3270 (3)	0.074 (2)	
H6A	0.3657	0.6523	0.3624	0.089*	
C7	0.5039 (7)	0.6426 (7)	0.3203 (3)	0.088 (3)	
H7A	0.5490	0.6474	0.3512	0.106*	
C8	0.5444 (7)	0.6337 (6)	0.2682 (4)	0.080 (3)	
H8A	0.6178	0.6304	0.2627	0.096*	
C9	0.4753 (6)	0.6294 (6)	0.2234 (3)	0.069 (2)	
H9A	0.5041	0.6265	0.1874	0.083*	
C10	−0.1049 (6)	0.5672 (5)	0.3110 (3)	0.069 (2)	
C11	0.3254 (5)	0.6217 (5)	0.0158 (3)	0.0561 (18)	
C12	0.3730 (6)	0.6163 (7)	−0.0363 (3)	0.081 (3)	
H12A	0.3285	0.6133	−0.0675	0.098*	
C13	0.5371 (6)	0.6203 (6)	0.0005 (3)	0.067 (2)	
C14	0.4929 (6)	0.6226 (7)	0.0529 (3)	0.086 (3)	
H14A	0.5377	0.6227	0.0840	0.103*	
C15	0.2073 (6)	0.6224 (5)	0.0227 (3)	0.0508 (16)	
C16	0.1397 (7)	0.6286 (7)	−0.0234 (3)	0.084 (3)	
H16A	0.1672	0.6347	−0.0594	0.100*	
C17	0.0316 (6)	0.6256 (8)	−0.0145 (3)	0.084 (3)	
H17A	−0.0151	0.6285	−0.0448	0.101*	
C18	−0.0068 (6)	0.6185 (6)	0.0383 (3)	0.074 (2)	
H18A	−0.0799	0.6163	0.0452	0.089*	
C19	0.0659 (6)	0.6145 (7)	0.0814 (3)	0.074 (2)	
H19A	0.0394	0.6100	0.1177	0.089*	
C20	0.6515 (6)	0.6222 (7)	−0.0092 (3)	0.079 (2)	
C21	0.2075 (5)	0.3957 (4)	0.1521 (3)	0.0519 (14)	
C22	0.2084 (5)	0.2977 (5)	0.1513 (4)	0.0630 (17)	
F1	0.1244 (6)	0.2588 (4)	0.1377 (5)	0.237 (7)	
F2	0.2200 (12)	0.2641 (5)	0.1986 (3)	0.257 (8)	
F3	0.2781 (8)	0.2573 (4)	0.1263 (5)	0.224 (6)	
Ag2	0.28673 (4)	−0.10754 (3)	0.34638 (2)	0.05706 (16)	
N9	0.1721 (5)	−0.1347 (5)	0.4381 (2)	0.074 (2)	
N10	0.0807 (5)	−0.1065 (5)	0.5421 (2)	0.0752 (19)	
N11	0.3892 (5)	−0.1344 (5)	0.4241 (2)	0.0633 (18)	
N12	−0.1796 (6)	−0.1231 (8)	0.5156 (4)	0.124 (3)	
N13	0.4147 (4)	−0.1294 (4)	0.2578 (2)	0.0556 (15)	
N14	0.5064 (5)	−0.1172 (7)	0.1529 (3)	0.105 (3)	
N15	0.1944 (4)	−0.1402 (4)	0.2678 (2)	0.0504 (13)	
N16	0.7638 (6)	−0.0743 (5)	0.1827 (3)	0.0792 (19)	
O3	0.3252 (4)	0.0539 (4)	0.3587 (3)	0.0792 (17)	
O4	0.1491 (4)	0.0458 (4)	0.3452 (3)	0.0895 (17)	
C23	0.0680 (6)	−0.1309 (7)	0.4448 (3)	0.086 (3)	
H23A	0.0237	−0.1366	0.4137	0.104*	
C24	0.0229 (5)	−0.1187 (6)	0.4971 (3)	0.0627 (19)	
C25	0.1865 (6)	−0.1075 (6)	0.5344 (3)	0.071 (2)	
H25A	0.2307	−0.0969	0.5650	0.085*	
C26	0.2328 (6)	−0.1237 (5)	0.4826 (3)	0.0562 (17)	
C27	0.3500 (6)	−0.1277 (6)	0.4758 (3)	0.0569 (19)	
C28	0.4188 (6)	−0.1227 (6)	0.5214 (3)	0.069 (2)	
H28A	0.3916	−0.1190	0.5576	0.083*	
C29	0.5277 (6)	−0.1233 (7)	0.5126 (3)	0.081 (3)	
H29A	0.5740	−0.1185	0.5429	0.097*	
C30	0.5669 (6)	−0.1308 (7)	0.4603 (3)	0.082 (3)	
H30A	0.6401	−0.1321	0.4537	0.099*	
C31	0.4952 (6)	−0.1365 (7)	0.4169 (3)	0.078 (3)	
H31A	0.5215	−0.1420	0.3806	0.094*	
C32	−0.0910 (6)	−0.1207 (7)	0.5061 (3)	0.083 (3)	
C33	0.3560 (5)	−0.1388 (4)	0.2121 (2)	0.0450 (15)	
C34	0.4038 (6)	−0.1342 (9)	0.1600 (4)	0.105 (4)	
H34A	0.3618	−0.1434	0.1284	0.126*	
C35	0.5629 (5)	−0.1076 (5)	0.1990 (3)	0.0567 (17)	
C36	0.5175 (5)	−0.1122 (5)	0.2514 (3)	0.0600 (18)	
H36A	0.5599	−0.1032	0.2829	0.072*	
C37	0.2388 (5)	−0.1545 (4)	0.2175 (2)	0.0451 (14)	
C38	0.1781 (6)	−0.1766 (5)	0.1720 (3)	0.0629 (19)	
H38A	0.2100	−0.1889	0.1377	0.075*	
C39	0.0679 (7)	−0.1805 (6)	0.1779 (3)	0.076 (2)	
H39A	0.0254	−0.1951	0.1472	0.091*	
C40	0.0224 (6)	−0.1632 (6)	0.2279 (3)	0.072 (2)	
H40A	−0.0514	−0.1640	0.2322	0.087*	
C41	0.0882 (5)	−0.1442 (5)	0.2721 (3)	0.0624 (19)	
H41A	0.0574	−0.1337	0.3070	0.075*	
C42	0.6758 (6)	−0.0875 (5)	0.1905 (3)	0.0636 (18)	
C43	0.2342 (5)	0.0832 (5)	0.3523 (3)	0.0583 (16)	
C44	0.2298 (6)	0.1823 (5)	0.3488 (4)	0.0670 (18)	
F4	0.1610 (7)	0.2222 (5)	0.3759 (5)	0.224 (6)	
F5	0.2152 (12)	0.2131 (5)	0.3023 (3)	0.271 (9)	
F6	0.3113 (6)	0.2237 (4)	0.3644 (5)	0.228 (6)	

### Atomic displacement parameters (Å^2^)

**Table:**

	*U*^11^	*U*^22^	*U*^33^	*U*^12^	*U*^13^	*U*^23^
Ag1	0.0607 (3)	0.0771 (3)	0.0347 (2)	−0.0027 (2)	−0.0086 (2)	−0.0001 (2)
N1	0.049 (3)	0.063 (3)	0.043 (3)	−0.001 (3)	0.000 (2)	0.002 (3)
N2	0.062 (4)	0.110 (5)	0.056 (4)	−0.006 (4)	0.013 (4)	−0.016 (4)
N3	0.049 (3)	0.072 (4)	0.037 (3)	−0.007 (3)	−0.006 (2)	−0.002 (3)
N4	0.072 (5)	0.091 (5)	0.115 (6)	−0.010 (4)	0.031 (4)	−0.019 (4)
N5	0.050 (4)	0.153 (7)	0.035 (3)	−0.003 (4)	−0.003 (3)	0.000 (4)
N6	0.058 (4)	0.145 (7)	0.052 (4)	0.006 (4)	0.003 (3)	0.014 (4)
N7	0.051 (3)	0.094 (5)	0.036 (3)	−0.006 (3)	0.000 (2)	0.010 (3)
N8	0.047 (4)	0.177 (9)	0.103 (6)	−0.003 (5)	0.007 (4)	−0.001 (6)
O1	0.047 (3)	0.068 (3)	0.083 (3)	−0.001 (2)	−0.003 (3)	−0.002 (3)
O2	0.049 (3)	0.077 (3)	0.097 (4)	0.011 (2)	−0.007 (3)	−0.002 (4)
C1	0.051 (4)	0.070 (4)	0.044 (4)	−0.007 (4)	0.003 (3)	−0.006 (3)
C2	0.048 (4)	0.065 (4)	0.055 (4)	0.001 (3)	0.007 (3)	0.002 (3)
C3	0.071 (5)	0.106 (6)	0.040 (4)	−0.003 (4)	0.006 (4)	−0.014 (4)
C4	0.059 (4)	0.051 (4)	0.041 (3)	−0.002 (4)	0.007 (3)	0.000 (3)
C5	0.055 (4)	0.063 (4)	0.035 (3)	−0.011 (3)	−0.002 (3)	−0.001 (3)
C6	0.078 (6)	0.101 (6)	0.044 (4)	−0.026 (5)	−0.009 (4)	−0.005 (4)
C7	0.057 (5)	0.151 (9)	0.057 (5)	−0.030 (5)	−0.023 (4)	0.016 (5)
C8	0.056 (5)	0.116 (7)	0.068 (5)	−0.011 (5)	−0.018 (4)	0.001 (5)
C9	0.050 (4)	0.101 (6)	0.056 (4)	−0.016 (4)	0.003 (3)	0.004 (4)
C10	0.058 (5)	0.080 (5)	0.067 (5)	−0.005 (4)	0.016 (4)	−0.012 (4)
C11	0.050 (4)	0.076 (5)	0.042 (3)	−0.004 (4)	−0.006 (3)	0.013 (3)
C12	0.056 (5)	0.143 (8)	0.045 (4)	0.006 (5)	−0.005 (3)	0.017 (5)
C13	0.048 (4)	0.097 (6)	0.056 (5)	−0.008 (4)	−0.005 (3)	0.013 (4)
C14	0.051 (4)	0.157 (9)	0.049 (4)	−0.003 (5)	−0.011 (4)	0.004 (5)
C15	0.053 (4)	0.059 (4)	0.040 (3)	−0.004 (4)	−0.003 (3)	0.004 (3)
C16	0.071 (5)	0.144 (9)	0.037 (4)	−0.009 (5)	−0.002 (3)	0.014 (5)
C17	0.052 (4)	0.155 (10)	0.045 (4)	−0.005 (5)	−0.013 (3)	0.016 (5)
C18	0.051 (4)	0.115 (7)	0.056 (4)	0.001 (4)	−0.007 (3)	0.002 (5)
C19	0.051 (4)	0.128 (7)	0.045 (4)	0.009 (5)	−0.004 (3)	0.013 (5)
C20	0.055 (5)	0.119 (7)	0.063 (5)	0.004 (5)	0.000 (4)	0.010 (5)
C21	0.039 (3)	0.070 (4)	0.047 (3)	0.008 (3)	0.001 (3)	−0.004 (4)
C22	0.044 (4)	0.070 (4)	0.075 (5)	0.005 (3)	0.011 (4)	0.008 (4)
F1	0.145 (6)	0.068 (4)	0.50 (2)	−0.009 (4)	−0.149 (10)	−0.026 (7)
F2	0.54 (3)	0.081 (4)	0.147 (7)	0.007 (9)	−0.076 (12)	0.029 (4)
F3	0.208 (9)	0.073 (4)	0.390 (15)	0.004 (5)	0.164 (10)	−0.035 (6)
Ag2	0.0630 (3)	0.0748 (3)	0.0334 (2)	0.0014 (2)	−0.0081 (2)	−0.0026 (2)
N9	0.052 (4)	0.127 (6)	0.042 (3)	−0.008 (4)	−0.005 (3)	0.003 (3)
N10	0.059 (4)	0.124 (6)	0.043 (3)	0.013 (4)	0.010 (3)	0.008 (4)
N11	0.049 (3)	0.107 (5)	0.034 (3)	0.005 (3)	−0.003 (2)	0.009 (3)
N12	0.057 (5)	0.216 (11)	0.098 (6)	−0.009 (6)	−0.009 (4)	0.005 (7)
N13	0.045 (3)	0.079 (4)	0.042 (3)	−0.009 (3)	−0.003 (2)	−0.005 (3)
N14	0.049 (3)	0.219 (9)	0.046 (3)	−0.011 (5)	0.008 (3)	0.002 (6)
N15	0.040 (3)	0.073 (4)	0.038 (3)	−0.001 (3)	−0.005 (2)	−0.005 (2)
N16	0.055 (4)	0.086 (5)	0.096 (5)	−0.012 (4)	0.012 (4)	−0.010 (4)
O3	0.067 (3)	0.066 (3)	0.104 (5)	0.003 (3)	−0.018 (3)	−0.007 (3)
O4	0.059 (3)	0.100 (4)	0.110 (5)	−0.016 (3)	0.000 (4)	−0.022 (4)
C23	0.047 (4)	0.160 (10)	0.051 (5)	−0.011 (5)	−0.011 (4)	−0.004 (5)
C24	0.048 (4)	0.087 (5)	0.053 (4)	0.006 (4)	0.009 (3)	0.011 (4)
C25	0.055 (4)	0.117 (6)	0.040 (4)	0.005 (4)	0.001 (3)	−0.006 (4)
C26	0.059 (4)	0.066 (4)	0.044 (3)	−0.001 (4)	−0.010 (3)	0.009 (3)
C27	0.056 (4)	0.080 (5)	0.035 (4)	−0.002 (4)	−0.003 (3)	0.004 (3)
C28	0.060 (4)	0.108 (7)	0.040 (4)	−0.001 (4)	−0.007 (3)	0.009 (4)
C29	0.054 (4)	0.140 (9)	0.049 (4)	−0.003 (5)	−0.012 (4)	−0.001 (5)
C30	0.050 (4)	0.140 (8)	0.057 (5)	−0.001 (5)	−0.001 (4)	0.012 (5)
C31	0.058 (5)	0.132 (8)	0.045 (4)	0.002 (5)	0.007 (3)	0.004 (4)
C32	0.055 (5)	0.129 (8)	0.064 (5)	0.006 (5)	0.006 (4)	0.006 (5)
C33	0.043 (3)	0.058 (4)	0.034 (3)	0.003 (3)	−0.004 (3)	−0.003 (3)
C34	0.053 (4)	0.218 (12)	0.045 (4)	−0.008 (6)	−0.003 (4)	−0.016 (7)
C35	0.043 (3)	0.070 (4)	0.058 (4)	0.006 (3)	0.002 (3)	0.001 (4)
C36	0.054 (4)	0.080 (5)	0.046 (4)	−0.007 (4)	−0.005 (3)	−0.008 (4)
C37	0.045 (3)	0.056 (3)	0.034 (3)	0.001 (3)	−0.003 (3)	−0.004 (2)
C38	0.060 (4)	0.090 (5)	0.039 (4)	−0.013 (4)	−0.005 (3)	−0.001 (3)
C39	0.068 (5)	0.101 (6)	0.057 (5)	−0.026 (5)	−0.017 (4)	0.002 (4)
C40	0.045 (4)	0.098 (6)	0.074 (6)	−0.012 (4)	−0.017 (4)	0.009 (5)
C41	0.043 (4)	0.090 (6)	0.054 (4)	0.003 (4)	0.002 (3)	−0.003 (4)
C42	0.056 (4)	0.074 (5)	0.061 (4)	0.001 (4)	0.009 (3)	−0.009 (4)
C43	0.050 (4)	0.075 (4)	0.050 (4)	0.005 (3)	−0.005 (3)	−0.003 (3)
C44	0.050 (4)	0.076 (5)	0.075 (5)	0.008 (4)	−0.002 (5)	0.002 (5)
F4	0.208 (9)	0.101 (5)	0.363 (14)	0.032 (5)	0.175 (10)	−0.015 (6)
F5	0.58 (3)	0.102 (5)	0.135 (7)	0.060 (11)	−0.064 (12)	0.023 (5)
F6	0.152 (7)	0.067 (4)	0.464 (18)	0.001 (4)	−0.135 (10)	−0.016 (7)

### Geometric parameters (Å, º)

**Table:**

Ag1—N7	2.272 (5)	Ag2—N15	2.254 (5)
Ag1—N3	2.295 (5)	Ag2—N11	2.290 (5)
Ag1—O1	2.425 (5)	Ag2—O3	2.481 (5)
Ag1—N1	2.552 (5)	N9—C23	1.315 (10)
N1—C4	1.317 (8)	N9—C26	1.317 (9)
N1—C1	1.337 (8)	N10—C24	1.307 (9)
N2—C3	1.322 (10)	N10—C25	1.338 (9)
N2—C2	1.349 (9)	N11—C27	1.330 (8)
N3—C9	1.317 (9)	N11—C31	1.338 (9)
N3—C5	1.342 (8)	N12—C32	1.133 (10)
N4—C10	1.138 (9)	N13—C36	1.322 (8)
N5—C11	1.323 (9)	N13—C33	1.322 (8)
N5—C14	1.335 (9)	N14—C35	1.316 (9)
N6—C12	1.310 (9)	N14—C34	1.320 (10)
N6—C13	1.331 (9)	N15—C41	1.335 (8)
N7—C19	1.298 (9)	N15—C37	1.340 (7)
N7—C15	1.340 (8)	N16—C42	1.135 (9)
N8—C20	1.125 (9)	O3—C43	1.231 (8)
O1—C21	1.236 (7)	O4—C43	1.217 (8)
O2—C21	1.228 (7)	C23—C24	1.380 (10)
C1—C2	1.383 (9)	C23—H23A	0.9300
C1—H1A	0.9300	C24—C32	1.443 (10)
C2—C10	1.443 (10)	C25—C26	1.386 (9)
C3—C4	1.390 (9)	C25—H25A	0.9300
C3—H3A	0.9300	C26—C27	1.478 (10)
C4—C5	1.505 (10)	C27—C28	1.389 (9)
C5—C6	1.373 (9)	C28—C29	1.379 (10)
C6—C7	1.368 (12)	C28—H28A	0.9300
C6—H6A	0.9300	C29—C30	1.346 (10)
C7—C8	1.348 (11)	C29—H29A	0.9300
C7—H7A	0.9300	C30—C31	1.374 (10)
C8—C9	1.378 (10)	C30—H30A	0.9300
C8—H8A	0.9300	C31—H31A	0.9300
C9—H9A	0.9300	C33—C34	1.382 (10)
C11—C12	1.381 (10)	C33—C37	1.492 (9)
C11—C15	1.487 (9)	C34—H34A	0.9300
C12—H12A	0.9300	C35—C36	1.374 (9)
C13—C14	1.366 (10)	C35—C42	1.460 (10)
C13—C20	1.451 (10)	C36—H36A	0.9300
C14—H14A	0.9300	C37—C38	1.366 (8)
C15—C16	1.391 (10)	C38—C39	1.388 (11)
C16—C17	1.370 (11)	C38—H38A	0.9300
C16—H16A	0.9300	C39—C40	1.347 (11)
C17—C18	1.353 (10)	C39—H39A	0.9300
C17—H17A	0.9300	C40—C41	1.368 (10)
C18—C19	1.375 (10)	C40—H40A	0.9300
C18—H18A	0.9300	C41—H41A	0.9300
C19—H19A	0.9300	C43—C44	1.486 (10)
C21—C22	1.467 (10)	C44—F5	1.215 (9)
C22—F3	1.218 (8)	C44—F4	1.231 (9)
C22—F2	1.243 (9)	C44—F6	1.251 (9)
C22—F1	1.245 (8)		
			
N7—Ag1—N3	154.8 (2)	F2—C22—C21	113.1 (8)
N7—Ag1—O1	106.2 (2)	F1—C22—C21	117.6 (6)
N3—Ag1—O1	98.7 (2)	N15—Ag2—N11	157.2 (2)
N7—Ag1—N1	111.66 (17)	N15—Ag2—O3	114.1 (2)
N3—Ag1—N1	68.41 (17)	N11—Ag2—O3	88.1 (2)
O1—Ag1—N1	97.50 (19)	C23—N9—C26	117.9 (7)
C4—N1—C1	118.1 (6)	C24—N10—C25	115.8 (6)
C4—N1—Ag1	113.5 (4)	C27—N11—C31	119.2 (6)
C1—N1—Ag1	128.5 (4)	C27—N11—Ag2	122.0 (5)
C3—N2—C2	115.1 (6)	C31—N11—Ag2	117.1 (4)
C9—N3—C5	117.3 (6)	C36—N13—C33	117.9 (6)
C9—N3—Ag1	118.7 (5)	C35—N14—C34	115.9 (7)
C5—N3—Ag1	122.6 (4)	C41—N15—C37	118.3 (6)
C11—N5—C14	117.8 (6)	C41—N15—Ag2	117.2 (4)
C12—N6—C13	115.7 (7)	C37—N15—Ag2	124.5 (4)
C19—N7—C15	118.0 (6)	C43—O3—Ag2	98.8 (4)
C19—N7—Ag1	116.3 (4)	N9—C23—C24	121.5 (7)
C15—N7—Ag1	125.1 (5)	N9—C23—H23A	119.3
C21—O1—Ag1	105.4 (4)	C24—C23—H23A	119.3
N1—C1—C2	120.9 (6)	N10—C24—C23	122.2 (6)
N1—C1—H1A	119.5	N10—C24—C32	115.3 (6)
C2—C1—H1A	119.5	C23—C24—C32	122.4 (7)
N2—C2—C1	122.0 (6)	N10—C25—C26	122.6 (6)
N2—C2—C10	115.6 (6)	N10—C25—H25A	118.7
C1—C2—C10	122.4 (7)	C26—C25—H25A	118.7
N2—C3—C4	123.7 (7)	N9—C26—C25	119.9 (7)
N2—C3—H3A	118.1	N9—C26—C27	118.7 (6)
C4—C3—H3A	118.1	C25—C26—C27	121.4 (6)
N1—C4—C3	120.1 (7)	N11—C27—C28	120.0 (7)
N1—C4—C5	118.2 (6)	N11—C27—C26	118.2 (6)
C3—C4—C5	121.1 (6)	C28—C27—C26	121.8 (6)
N3—C5—C6	122.1 (7)	C29—C28—C27	119.6 (7)
N3—C5—C4	116.2 (6)	C29—C28—H28A	120.2
C6—C5—C4	121.7 (6)	C27—C28—H28A	120.2
C5—C6—C7	119.3 (8)	C30—C29—C28	120.1 (7)
C5—C6—H6A	120.4	C30—C29—H29A	119.9
C7—C6—H6A	120.4	C28—C29—H29A	119.9
C8—C7—C6	118.9 (7)	C29—C30—C31	117.7 (7)
C8—C7—H7A	120.6	C29—C30—H30A	121.1
C6—C7—H7A	120.6	C31—C30—H30A	121.1
C7—C8—C9	119.0 (8)	N11—C31—C30	123.3 (7)
C7—C8—H8A	120.5	N11—C31—H31A	118.3
C9—C8—H8A	120.5	C30—C31—H31A	118.3
N3—C9—C8	123.3 (7)	N12—C32—C24	176.9 (10)
N3—C9—H9A	118.3	N13—C33—C34	119.7 (6)
C8—C9—H9A	118.3	N13—C33—C37	119.5 (5)
N4—C10—C2	177.1 (9)	C34—C33—C37	120.8 (6)
N5—C11—C12	118.5 (6)	N14—C34—C33	123.2 (8)
N5—C11—C15	119.6 (6)	N14—C34—H34A	118.4
C12—C11—C15	121.9 (6)	C33—C34—H34A	118.4
N6—C12—C11	124.8 (7)	N14—C35—C36	122.2 (6)
N6—C12—H12A	117.6	N14—C35—C42	115.3 (6)
C11—C12—H12A	117.6	C36—C35—C42	122.5 (6)
N6—C13—C14	121.0 (7)	N13—C36—C35	121.1 (6)
N6—C13—C20	115.9 (7)	N13—C36—H36A	119.4
C14—C13—C20	123.1 (7)	C35—C36—H36A	119.4
N5—C14—C13	122.0 (7)	N15—C37—C38	121.2 (6)
N5—C14—H14A	119.0	N15—C37—C33	117.4 (5)
C13—C14—H14A	119.0	C38—C37—C33	121.2 (6)
N7—C15—C16	121.3 (7)	C37—C38—C39	119.0 (7)
N7—C15—C11	117.4 (6)	C37—C38—H38A	120.5
C16—C15—C11	121.3 (6)	C39—C38—H38A	120.5
C17—C16—C15	118.5 (7)	C40—C39—C38	120.1 (7)
C17—C16—H16A	120.7	C40—C39—H39A	120.0
C15—C16—H16A	120.7	C38—C39—H39A	120.0
C18—C17—C16	119.9 (7)	C39—C40—C41	117.9 (7)
C18—C17—H17A	120.1	C39—C40—H40A	121.1
C16—C17—H17A	120.1	C41—C40—H40A	121.1
C17—C18—C19	117.6 (7)	N15—C41—C40	123.4 (7)
C17—C18—H18A	121.2	N15—C41—H41A	118.3
C19—C18—H18A	121.2	C40—C41—H41A	118.3
N7—C19—C18	124.6 (7)	N16—C42—C35	177.6 (9)
N7—C19—H19A	117.7	O4—C43—O3	131.6 (7)
C18—C19—H19A	117.7	O4—C43—C44	114.8 (6)
N8—C20—C13	177.4 (9)	O3—C43—C44	113.4 (6)
O2—C21—O1	129.9 (7)	F5—C44—F4	101.0 (8)
O2—C21—C22	116.2 (6)	F5—C44—F6	101.9 (9)
O1—C21—C22	113.8 (5)	F4—C44—F6	100.1 (8)
F3—C22—F2	99.2 (8)	F5—C44—C43	115.7 (8)
F3—C22—F1	104.2 (9)	F4—C44—C43	118.8 (8)
F2—C22—F1	98.4 (8)	F6—C44—C43	116.5 (7)
F3—C22—C21	120.5 (7)		
			
N7—Ag1—N1—C4	−149.5 (5)	Ag1—O1—C21—C22	178.3 (5)
N3—Ag1—N1—C4	3.3 (5)	O2—C21—C22—F3	142.7 (10)
O1—Ag1—N1—C4	99.7 (5)	O1—C21—C22—F3	−38.0 (13)
N7—Ag1—N1—C1	31.1 (6)	O2—C21—C22—F2	−100.3 (11)
N3—Ag1—N1—C1	−176.0 (7)	O1—C21—C22—F2	79.0 (11)
O1—Ag1—N1—C1	−79.7 (6)	O2—C21—C22—F1	13.5 (13)
N7—Ag1—N3—C9	−94.7 (7)	O1—C21—C22—F1	−167.2 (10)
O1—Ag1—N3—C9	75.4 (6)	N15—Ag2—N11—C27	−109.2 (7)
N1—Ag1—N3—C9	170.1 (7)	O3—Ag2—N11—C27	83.7 (7)
N7—Ag1—N3—C5	98.8 (7)	N15—Ag2—N11—C31	86.1 (9)
O1—Ag1—N3—C5	−91.1 (6)	O3—Ag2—N11—C31	−81.0 (7)
N1—Ag1—N3—C5	3.6 (5)	N11—Ag2—N15—C41	101.2 (7)
N3—Ag1—N7—C19	−100.3 (8)	O3—Ag2—N15—C41	−93.0 (6)
O1—Ag1—N7—C19	90.0 (7)	N11—Ag2—N15—C37	−79.6 (7)
N1—Ag1—N7—C19	−15.2 (7)	O3—Ag2—N15—C37	86.2 (5)
N3—Ag1—N7—C15	89.0 (8)	N15—Ag2—O3—C43	51.4 (6)
O1—Ag1—N7—C15	−80.8 (6)	N11—Ag2—O3—C43	−134.1 (5)
N1—Ag1—N7—C15	174.1 (6)	C26—N9—C23—C24	−2.0 (15)
N7—Ag1—O1—C21	−53.2 (5)	C25—N10—C24—C23	−0.6 (14)
N3—Ag1—O1—C21	131.2 (5)	C25—N10—C24—C32	177.8 (9)
N1—Ag1—O1—C21	62.0 (5)	N9—C23—C24—N10	2.9 (16)
C4—N1—C1—C2	−1.2 (11)	N9—C23—C24—C32	−175.4 (10)
Ag1—N1—C1—C2	178.1 (5)	C24—N10—C25—C26	−2.4 (13)
C3—N2—C2—C1	−4.0 (12)	C23—N9—C26—C25	−0.9 (13)
C3—N2—C2—C10	176.3 (8)	C23—N9—C26—C27	−180.0 (9)
N1—C1—C2—N2	4.1 (12)	N10—C25—C26—N9	3.3 (13)
N1—C1—C2—C10	−176.2 (7)	N10—C25—C26—C27	−177.7 (8)
C2—N2—C3—C4	1.4 (13)	C31—N11—C27—C28	0.2 (13)
C1—N1—C4—C3	−1.4 (11)	Ag2—N11—C27—C28	−164.2 (6)
Ag1—N1—C4—C3	179.2 (6)	C31—N11—C27—C26	178.8 (8)
C1—N1—C4—C5	170.4 (6)	Ag2—N11—C27—C26	14.4 (11)
Ag1—N1—C4—C5	−9.1 (8)	N9—C26—C27—N11	5.8 (13)
N2—C3—C4—N1	1.4 (13)	C25—C26—C27—N11	−173.2 (8)
N2—C3—C4—C5	−170.1 (8)	N9—C26—C27—C28	−175.6 (8)
C9—N3—C5—C6	2.5 (11)	C25—C26—C27—C28	5.3 (13)
Ag1—N3—C5—C6	169.2 (6)	N11—C27—C28—C29	1.0 (14)
C9—N3—C5—C4	−176.1 (7)	C26—C27—C28—C29	−177.5 (9)
Ag1—N3—C5—C4	−9.4 (9)	C27—C28—C29—C30	−1.6 (16)
N1—C4—C5—N3	12.4 (10)	C28—C29—C30—C31	0.9 (16)
C3—C4—C5—N3	−175.9 (7)	C27—N11—C31—C30	−0.9 (15)
N1—C4—C5—C6	−166.2 (7)	Ag2—N11—C31—C30	164.2 (8)
C3—C4—C5—C6	5.4 (12)	C29—C30—C31—N11	0.4 (16)
N3—C5—C6—C7	−4.0 (13)	C36—N13—C33—C34	2.6 (12)
C4—C5—C6—C7	174.6 (8)	C36—N13—C33—C37	−177.5 (7)
C5—C6—C7—C8	1.6 (15)	C35—N14—C34—C33	2.2 (17)
C6—C7—C8—C9	1.9 (15)	N13—C33—C34—N14	−2.7 (17)
C5—N3—C9—C8	1.2 (13)	C37—C33—C34—N14	177.5 (10)
Ag1—N3—C9—C8	−166.0 (7)	C34—N14—C35—C36	−1.9 (15)
C7—C8—C9—N3	−3.4 (15)	C34—N14—C35—C42	−179.0 (9)
C14—N5—C11—C12	0.3 (14)	C33—N13—C36—C35	−2.4 (11)
C14—N5—C11—C15	179.1 (9)	N14—C35—C36—N13	2.1 (13)
C13—N6—C12—C11	−0.5 (15)	C42—C35—C36—N13	179.0 (7)
N5—C11—C12—N6	−1.0 (15)	C41—N15—C37—C38	−2.8 (10)
C15—C11—C12—N6	−179.9 (9)	Ag2—N15—C37—C38	178.0 (5)
C12—N6—C13—C14	2.8 (15)	C41—N15—C37—C33	172.7 (6)
C12—N6—C13—C20	−178.0 (9)	Ag2—N15—C37—C33	−6.4 (8)
C11—N5—C14—C13	2.0 (16)	N13—C33—C37—N15	13.5 (10)
N6—C13—C14—N5	−3.7 (17)	C34—C33—C37—N15	−166.7 (8)
C20—C13—C14—N5	177.1 (10)	N13—C33—C37—C38	−170.9 (7)
C19—N7—C15—C16	1.8 (12)	C34—C33—C37—C38	8.9 (12)
Ag1—N7—C15—C16	172.4 (7)	N15—C37—C38—C39	2.7 (11)
C19—N7—C15—C11	−178.0 (8)	C33—C37—C38—C39	−172.7 (7)
Ag1—N7—C15—C11	−7.4 (10)	C37—C38—C39—C40	−0.4 (13)
N5—C11—C15—N7	−6.9 (13)	C38—C39—C40—C41	−1.6 (14)
C12—C11—C15—N7	171.9 (8)	C37—N15—C41—C40	0.6 (12)
N5—C11—C15—C16	173.4 (9)	Ag2—N15—C41—C40	179.9 (6)
C12—C11—C15—C16	−7.8 (13)	C39—C40—C41—N15	1.6 (13)
N7—C15—C16—C17	−2.2 (14)	Ag2—O3—C43—O4	3.7 (11)
C11—C15—C16—C17	177.6 (9)	Ag2—O3—C43—C44	−171.6 (6)
C15—C16—C17—C18	1.2 (16)	O4—C43—C44—F5	−71.1 (12)
C16—C17—C18—C19	0.0 (16)	O3—C43—C44—F5	105.0 (11)
C15—N7—C19—C18	−0.5 (15)	O4—C43—C44—F4	49.4 (13)
Ag1—N7—C19—C18	−171.9 (8)	O3—C43—C44—F4	−134.5 (10)
C17—C18—C19—N7	−0.4 (16)	O4—C43—C44—F6	169.2 (9)
Ag1—O1—C21—O2	−2.5 (10)	O3—C43—C44—F6	−14.7 (13)
